# The differentiation/retrodifferentiation program of human U937 leukemia cells is accompanied by changes of VCP/p97

**DOI:** 10.1186/1471-2121-9-12

**Published:** 2008-02-15

**Authors:** Catharina Bertram, Nils von Neuhoff, Britta Skawran, Doris Steinemann, Brigitte Schlegelberger, Ralf Hass

**Affiliations:** 1 Dept. of Gynecology, Biochemistry and Tumor Biology Lab, Medical School Hannover, Carl-Neuberg-Str. 1, D - 30625 Hannover, Germany; 2 Institute of Cell and Molecular Pathology, Medical School, Hannover, Germany

## Abstract

**Background:**

Retrodifferentiation and regained proliferative capacity of growth-arrested human leukemic cells after monocyte-like differentiation requires proteolytic activities together with distinct regulatory factors. The AAA ATPase valosin-containing protein (VCP/p97) contributes to protein degradation and cell cycle regulation, respectively, and it was of interest to study a possible role of VCP/p97 during this myelomonocytic differentiation and retrodifferentiation.

**Results:**

Separation of autonomously proliferating human U937 myeloid leukemia cells by centrifugal elutriation demonstrated unaltered VCP/p97 expression levels throughout distinct phases of the cell cycle. However, phorbol ester-induced G_0_/G_1 _cell cycle arrest in differentiating human U937 leukemia cells was associated with a significantly increased protein and mRNA amount of this AAA ATPase. These elevated VCP/p97 levels progressively decreased again when growth-arrested U937 cells entered a retrodifferentiation program and returned to the tumorigenic phenotype. Whereas VCP/p97 was observed predominantly in the cytosol of U937 tumor and retrodifferentiated cells, a significant nuclear accumulation appeared during differentiation and G_0_/G_1_ growth arrest. Analysis of subcellular compartments by immunoprecipitations and 2D Western blots substantiated these findings and revealed furthermore a tyrosine-specific phosphorylation of VCP/p97 in the cytosolic but not in the nuclear fractions. These altered tyrosine phosphorylation levels, according to distinct subcellular distributions, indicated a possible functional involvement of VCP/p97 in the leukemic differentiation process. Indeed, a down-modulation of VCP/p97 protein by siRNA revealed a reduced expression of differentiation-associated genes in subsequent DNA microarray analysis. Moreover, DNA-binding and proliferation-associated genes, which are down-regulated during differentiation of the leukemic cells, demonstrated elevated levels in the VCP/p97 siRNA transfectants.

**Conclusion:**

The findings demonstrated that monocytic differentiation and G_0_/G_1 _growth arrest in human U937 leukemia cells was accompanied by an increase in VCP/p97 expression and a distinct subcellular distribution to be reverted during retrodifferentiation. Together with a down-modulation of VCP/p97 by siRNA, these results suggested an association of this AAA ATPase in the differentiation/retrodifferentiation program.

## Background

The human myeloid leukemia cell line U937 represents a well-characterized *in vitro *tumor model of differentiation and retrodifferentiation, which can be induced to differentiate along the monocyte/macrophage-like pathway after treatment with 12-O-tetradecanoyl-phorbol-13-acetate (TPA) [1]. During this differentiation process, the autonomously proliferating tumor cells become adherent, undergo a complete growth arrest and acquire a variety of morphological and functional changes typical for normal monocytes and macrophages [1-5]. The TPA-differentiated U937 cells remain and survive in a transient G_0_' growth arrest cycle [6,7]. After 2–3 weeks in the absence of TPA, however, the differentiated population down-modulates all markers acquired during the previous differentiation process and re-enters the proliferative cell cycle by a retrodifferentiation program, which results in the undifferentiated phenotype [6-8]. Recent studies on the mechanism of this retrodifferentiation process revealed the necessity of a tightly regulated protein modulation program, which involves the cellular proteasome [9] and a variety of regulatory compounds, including the nuclear factor poly(ADP-ribose)polymerase [10]. However, since fundamental cellular functions are altered and reverted in this differentiation and retrodifferentiation model [11], the precise signaling pathways for the coordination of this phenomenon remain unclear and may thus involve further regulatory compounds of the proteasome, including certain ATPases.

The valosin-containing protein (VCP), also termed p97, belongs to the AAA superfamily (ATPases associated with a variety of cellular activities) and represents the mammalian homolog of Cdc48 in yeast [12]. VCP/p97 is one of the most abundant proteins in cells and is ubiquitously expressed in all eukaryotic cells. Thereby, it is involved in multiple cellular effects and performs a large variety of different cellular functions, including transport mechanisms, protein degradation and cell cycle regulation, respectively [13]. In this context, previous work has demonstrated that VCP/p97 targets numerous ubiquitinated substrates to the proteasome for subsequent degradation and may thus act as a general chaperone [14]. This concept is also reflected during cell cycle regulation [15], whereby VCP/p97 is associated with the degradation of decisive cell cycle regulatory proteins via the ubiquitin-proteasome pathway. For example, the mammalian cyclin E, which is required for the entry of cells into S phase [16], and the G_1_-CDK inhibitor Far1 are both affected in their stability by interference with VCP/p97 and subsequent proteasomal degradation [14,17]. However, the precise interaction of VCP/p97 and different signal cascades are unknown so far. Moreover, the activation process of VCP/p97 in distinct subcellular compartments and possible modifications, e.g. by phosphorylation, remains obscure.

VCP/p97 has been demonstrated to localize predominantly in the cytosol. However, according to its involvement in membrane fusion, this protein is also associated with various membranes of organelles such as the Golgi and the endoplasmic reticulum [18,19]. Nuclear localization of VCP/p97 was determined by interactions with Werner protein, a RecQ helicase [20], and during spindle disassembly [21]. Phosphorylation of VCP/p97 on tyrosine residues is suggested to play a role in determining the appropriate subcellular localization [15,22]. In addition, after DNA damage, VCP/p97 is phosphorylated at Ser784 [23]. Interestingly, the phosphorylation does not affect the VCP/p97 ATPase activity and therefore, the physiological relevance of these modifications remains unknown [24].

VCP/p97 has a variety of clinical implications and thus contributes to the pathogenesis of distinct human diseases. In this context, VCP/p97 plays a role in several neurodegenerative disorders, which may be associated with its chaperone-like function as a cell death effector molecule [25-27]. Furthermore, VCP/p97 is discussed to be involved in certain types of human tumors such as hepatocellular and colorectal carcinomas as well as breast cancer [13,27,28]. In addition, VCP/p97 may increase the metastatic potential in tumor entities, possibly by controlling the stability of IκBα, an inhibitor of NFκB [29,30]. Up to now the role of VCP/p97 during a cancer development is poorly understood. We therefore aim to characterize this ATPase in an *in vitro *cancer model of differentiation and retrodifferentiation to provide additional information for a potential role and function during tumorigenesis.

The predominant focus of the present study was 1) to examine the VCP/p97 expression in distinct cell cycle phases of proliferating U937 cells; 2) to analyze the regulation of VCP/p97 during differentiation-associated G_0_/G_1 _cell cycle exit and subsequent retrodifferentiation of U937 cells; 3) to investigate a possible functional association of VCP/p97 with the differentiation process.

## Results

**Figure 1:**
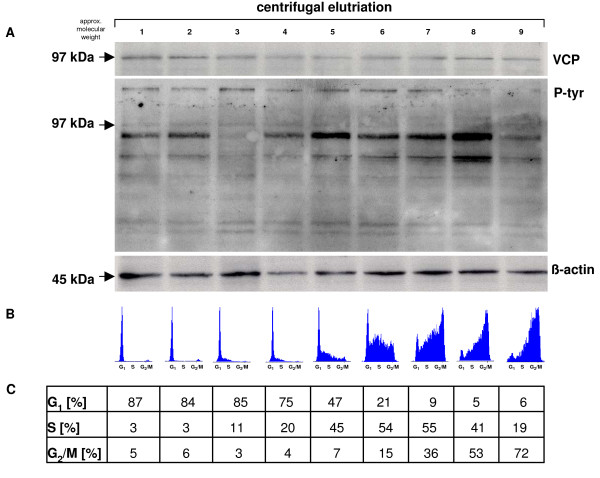
**VCP/p97 expression during cell cycle distribution of synchronized U937 cells**. **A**. U937 cells were separated by centrifugal elutriation and cell lysates of the appropriate fractions were analyzed by Western blot using an anti-VCP/p97 and an anti-phosphotyrosine antibody, respectively. Expression of β-actin was used as a loading control. **B**. The cell cycle distribution of the appropriate elutriated cell fractions were analyzed by flow cytometry and histograms were generated by the FloMax software. **C**. Quantification of the cell cycle distribution of the elutriated fractions and the percentage of the distinct cell cycle phases was gated and calculated using the MultiCycle software.

Previous studies have demonstrated an essential role for VCP/p97 in cell cycle regulation [14,17]. In order to investigate the expression of VCP/p97 during the cell cycle, centrifugal elutriations of proliferating U937 cells were performed to separate cells within distinct phases of the cell cycle (Fig. 1). Western blot analysis of the elutriated U937 cell lysates demonstrated little if any difference in the 97 kDa protein VCP/p97 levels throughout the cell cycle (Fig. 1A). Control of the elutriated cell cycle phases and quantification of the distribution was performed by flow cytometry (Fig. 1B, 1C). A tyrosine phosphorylation of an approximately 97 kDa protein was present in the G_1_-enriched populations and remained undetectable in S phase-enriched cells and during G_2_/M phase (Fig. 1). β-actin, which served as a loading control, was similarly expressed throughout the cell cycle (Fig. 1).

**Figure 2:**
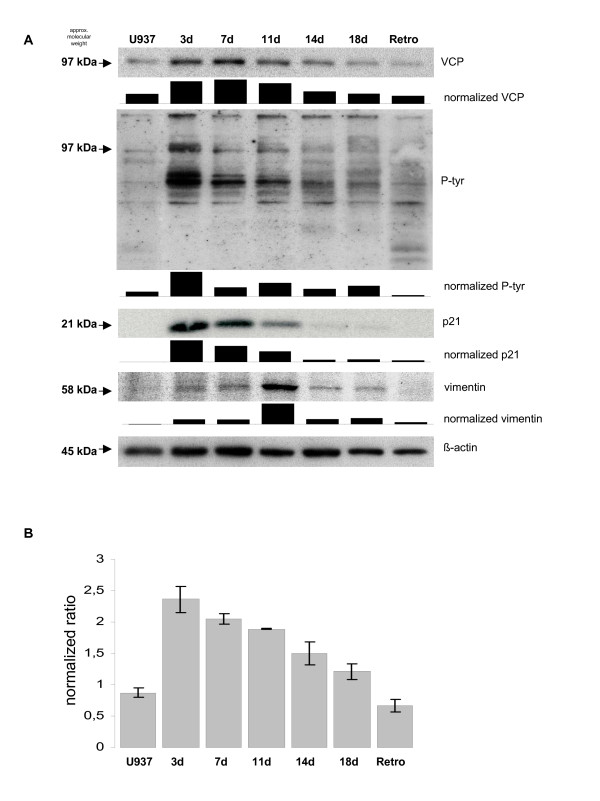
**Alteration of the VCP/p97 expression during differentiation and retrodifferentiation**. **A**. U937 cells were treated initially with 10 nM TPA and then cultured in the absence of TPA until retrodifferentiation. At the time points indicated, cell lysates were separated by 10% SDS-PAGE followed by Western blot analysis with anti-VCP/p97, anti-P-tyr, anti-p21^Cip1/Waf1/sdi-1 ^and anti-vimentin antibody. The tyrosine phosphorylation pattern (p-tyr) is demonstrated by the complete range from 250-20 kDa. Staining with β-actin was used as a loading control. Densitometric analysis and normalization to β-actin was performed using the ImageJ software (NIH, Bethesda, MD, USA). **B**. Quantitative RT-PCR analysis of VCP/p97 mRNA levels during differentiation and retrodifferentiation. Following total RNA isolation of undifferentiated U937 control cells (U937), differentiated populations (3d until 18d) and retrodifferentiated U937 cells (Retro), the amplification was performed in the LightCycler 2.0 System using the LightCycler Software 3.5. Results represent means ± SD from three independent experiments.

Next, we investigated VCP/p97 protein expression during growth arrest and differentiation. Treatment of U937 cells with 10 nM of the phorbol ester 12-O-tetradecanoyl-phorbol-13-acetate (TPA) for 72 h was associated with cell cycle arrest in G_0_' and subsequent differentiation along the monocytic/macrophage-like pathway [1,6]. Long term culture of these growth-arrested and differentiated cells in the absence of TPA for approximately 3 weeks was accompanied by a retrodifferentiation process, whereby the differentiated cells down-modulate all previously acquired differentiation markers and re-enter the proliferative cell cycle [6,7]. Indeed, TPA-induced differentiation of U937 cells demonstrated a significant expression of the cell cycle inhibitor p21^Cip1/Waf1/sdi-1 ^[31] within 3 d, which was down-regulated during retrodifferentiation (Fig. 2A). Simultaneously, the differentiation-associated intermediate filament protein vimentin [3] also exhibited marked expression during differentiation in contrast to a very low detection level in U937 control and retrodifferentiated cells (Fig. 2A). Expression of VCP/p97 was significantly elevated after 3 d of TPA exposure and this expression continuously decreased until the cells entered retrodifferentiation to reach the same VCP/p97 protein levels observed in untreated control cells (Fig. 2A). Similar findings were observed for tyrosine-phorphorylated proteins at approximately 97 kDa, demonstrating a marked presence after 3 d of TPA incubation and a progressive decline thereafter (Fig. 2A). Western blots were normalized to the β-actin levels by densitometry using the ImageJ software (NIH, Bethesda, MD, USA).

Following quantitative RT-PCR analysis of VCP/p97, the measurements were normalized to the uniformly transcribed mRNA of the TATA-binding protein (TBP). In agreement with the Western blot data, mRNA expression of VCP/p97 was significantly enhanced after 72 h of TPA induction and thereafter, the transcripts progressively decreased until the level of retrodifferentiated cells was indistinguishable to that observed in U937 control cells (Fig. 2B). Together, these data indicated a possible association of VCP/p97 with the differentiation process and the transient cell cycle arrest.

**Figure 3:**
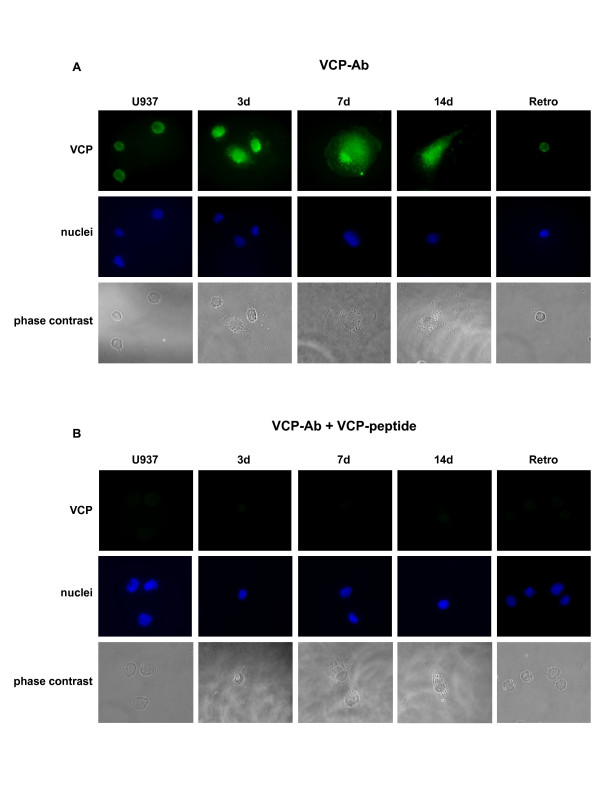
**Subcellular localization of VCP/p97 in undifferentiated controls (U937), differentiated populations (3d, 7d, 14d) and retrodifferentiated U937 cells (Retro) by immunofluorescence**. **A**. VCP/p97 was visualized by a secondary FITC-conjugated rabbit anti-goat antibody (VCP-Ab). The nuclei were stained with the DNA-intercalating dye Hoechst 33258. **B**. The VCP/p97 antibody was neutralized with a specific blocking peptide and incubated with a secondary FITC-conjugated rabbit anti-goat antibody (VCP-Ab + VCP-peptide). The nuclei were stained with the DNA-intercalating dye Hoechst 33258.

Phase contrast pictures clearly showed the distinct morphology of the differentiated U937 cells. Using staining with the DNA-intercalating dye Hoechst 33258, the appropriate compartments of the nuclei within these cells could easily be demonstrated (Fig. 3). Subcellular localization of VCP/p97 during the differentiation process was analyzed by immunofluorescence. Undifferentiated and retrodifferentiated U937 cells revealed a predominant localization of VCP/p97 in both, the cytoplasm and the plasma membrane, whereas there was less evidence for VCP/p97 localization in the nucleus of these cells (Fig. 3A). In contrast, the differentiated cell populations demonstrated opposite effects: A localization of VCP/p97 in the cytoplasm as well as a membrane association was paralleled by a pronounced immunofluorescence in the nucleus of all differentiated populations (Fig. 3A). The specificity of the VCP/p97 antibody was tested using an appropriate neutralizing peptide (Fig 3B). These data suggested that a significantly increased expression and an enhanced nuclear localization of VCP/p97 accompany differentiation and cell cycle arrest, which is reverted during the retrodifferentiation program.

**Figure 4:**
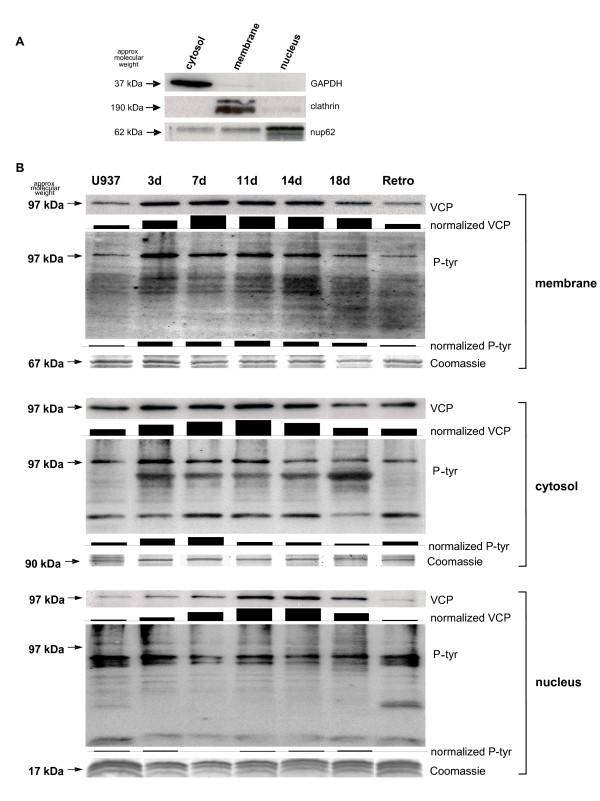
**Subcellular regulation of VCP/p97 during differentiation and retrodifferentiation**. **A**. The successful separation of the different U937 cell populations into subcellular compartments was controlled by GAPDH for the cytosol, clathrin for the membrane-fraction and nucleoporin62 (nup62) for the nucleus, respectively. **B**. Cytosol, membranes and nuclei of undifferentiated U937 control cells (U937), differentiated populations (3d until 18d) and retrodifferentiated U937 cells (Retro), respectively, were separated by SDS-PAGE, and VCP/p97 expression levels as well as the tyrosine phosphorylation patterns were examined by Western blot analysis. The Coomassie stain of the different fractions was used as a loading control. Densitometric analysis and normalization to the appropriate Coomassie stain was performed using the ImageJ software (NIH, Bethesda, MD, USA).

In order to validate these findings, distinct subcellular compartments were separated and analyzed by Western blotting (Fig. 4). Predominant expression of GAPDH in the cytosol, clathrin in the membrane-associated fraction, and nucleoporin62 in the nucleus, respectively, demonstrated a successful enrichment of the appropriate subcellular fractions (Fig. 4A). Western blot analysis revealed a different expression of VCP/p97 in distinct cellular compartments during differentiation and retrodifferentiation (Fig. 4B). Whereas little if any differences in the expression of VCP/p97 were detectable in the cytosol, VCP/p97 protein levels significantly increased in the membrane fraction after TPA induction, most predominantly at day 3, and returned back to control levels during the retrodifferentiation program (Fig 4B). Moreover, VCP/p97 expression in the nucleus was significantly elevated after TPA exposure most predominantly at day 11, and similarly returned to control levels at the time of retrodifferentiation (Fig. 4B). Whereby total amounts of VCP/p97 in the subcellular compartments were not quantified, an apparent inconsistency by comparison of these subcellular VCP distributions to the pattern observed in the time course of the total cell lysates (Fig. 2) may results from an unequal distribution, suggesting most of the VCP/p97 within the cytosol and membrane-associated as compared to the nuclear fraction.

Investigations of tyrosine phosphorylations within these subcellular compartments demonstrated a phosphorylation pattern of an approximately 97 kDa protein in the cytosol and the membranes similar to that observed for VCP/p97 in these compartments (Fig. 4B). In contrast, there was little if any tyrosine phosphorylation detectable at 97 kDa in the nucleus during differentiation and retrodifferentiation (Fig. 4B). Analysis of the Coomassie staining in the cytosol, membrane fraction and the nuclear compartment, respectively, served as an appropriate loading control. The protein levels were normalized to the Coomassie staining by densitometric analysis (Fig. 4B).

These data suggested a distinct subcellular distribution of VCP/p97 during the differentiation process and cell cycle arrest associated with tyrosine phosphorylation exclusively in the cytosol and membranes.

**Figure 5:**
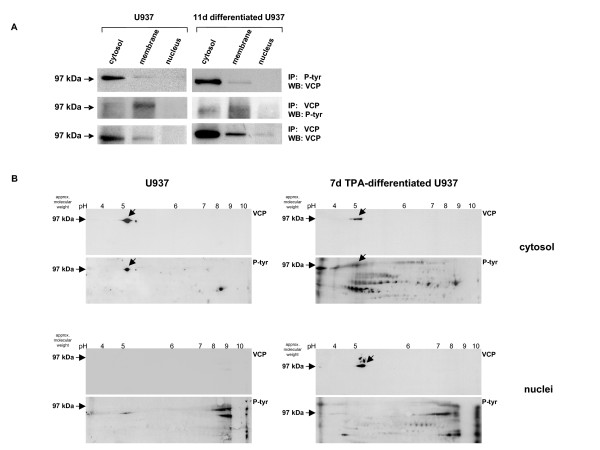
**Subcellular tyrosine-phosphorylation pattern of VCP/p97**. **A**. Immunoprecipitation of subcellular compartments during U937 differentiation and subsequent Western blot analysis. Cytosol, membranes, and nuclei of undifferentiated U937 control cells (U937) and 11d differentiated populations (TPA-differentiated U937), respectively, were immunoprecipitated with an anti-phosphotyrosine antibody (IP: P-tyr) followed by an anti-VCP/p97 Western blot (WB: VCP). A cross-immunoprecipitation was performed with anti-VCP/p97 (IP: VCP) and subsequent anti-phosphotyrosine Western blot (WB: P-tyr). Protein controls were evaluated by anti-VCP/p97 immunoprecipitation (IP: VCP) followed by anti-VCP/p97 Western blot (WB: VCP). **B**. 2D-Western blot analysis of cytosolic and nuclear fractions during U937 differentiation. Cytosolic and nuclear proteins from U937 control cells (U937) and 7d TPA-differentiated cells were separated by non-linear isoelectric focusing (pH 3–10, NL) and subsequent 10% SDS-PAGE in the second dimension. Western blot analysis was performed with an anti-VCP/p97 antibody (VCP) and after successful stripping, which revealed no detectable spots anymore (data not shown), the same blots were incubated with an anti-phosphotyrosine antibody (P-tyr) and developed by the ECL kit.

For characterization of these findings, we performed an anti-phosphotyrosine immunoprecipitation of the subcellular fractions in undifferentiated U937 and differentiated cells, exemplarily shown 11 days after TPA induction (Fig. 5A). VCP/p97 Western blot analysis of the phosphotyrosine-precipitated proteins revealed a distinct band in the molecular range of approximately 97 kDa in the cytosolic as well as in the membrane fractions of both, undifferentiated and differentiated cells. In contrast, little if any VCP/p97 was detectable in the nuclear fraction of either U937 control or differentiated cells (Fig. 5A). These data were supported by anti-VCP/p97 cross-immunoprecipitations followed by phosphotyrosine Western blots with a signal at approximately 97 kDa in the cytosolic and membraneous fractions of U937 and differentiated cells but no detectable phosphorylation in either nuclear fraction (Fig. 5A). Analysis of the protein amount in each fraction was performed by anti-VCP/p97 immunoprecitation and subsequent anti-VCP/p97 Western blot (Fig. 5A).

To further substantiate these findings, proteins of the cytosolic and nuclear compartments from undifferentiated and TPA-differentiated U937 cells were separated by non-linear isoelectric focusing (NL-IEF) and subsequent SDS-PAGE in the second dimension, respectively, followed by anti-VCP/p97 and anti-phosphotyrosine immunoblotting, respectively (Fig. 5B). The cytosolic 2D VCP/p97 Western blot of U937 and TPA-differentiated U937 cells demonstrated a distinct spot in the molecular range of 97 kDa and an isoelectric point of approximately pH 5.1 respectively (Fig. 5B). Stripping and rehybridization of these 2D blots with the anti-phosphotyrosine antibody revealed the same phosphorylated spots at about 97 kDa, pH 5.1, among a variety of other phosphorylated proteins (Fig. 5B). In the nuclear fraction of U937 cells, the 2D VCP/p97 Western blot could not visualize any protein spot and accordingly, no phosphorylation was detectable at about 97 kDa, pH 5.1 (Fig. 5B). In contrast, the nuclear fraction of TPA-differentiated U937 cells demonstrated a clear VCP/p97 protein spot at approximately 97 kDa, pH 5.1 and subsequent anti-phosphotyrosine Western blot did not reveal any phosphorylation of this protein spot (Fig. 5B), which is in line with our previous data. Control exposure of the stripped blots and control hybridizations with the secondary antibody alone revealed no detectable signals, respectively (data not shown).

**Figure 6:**
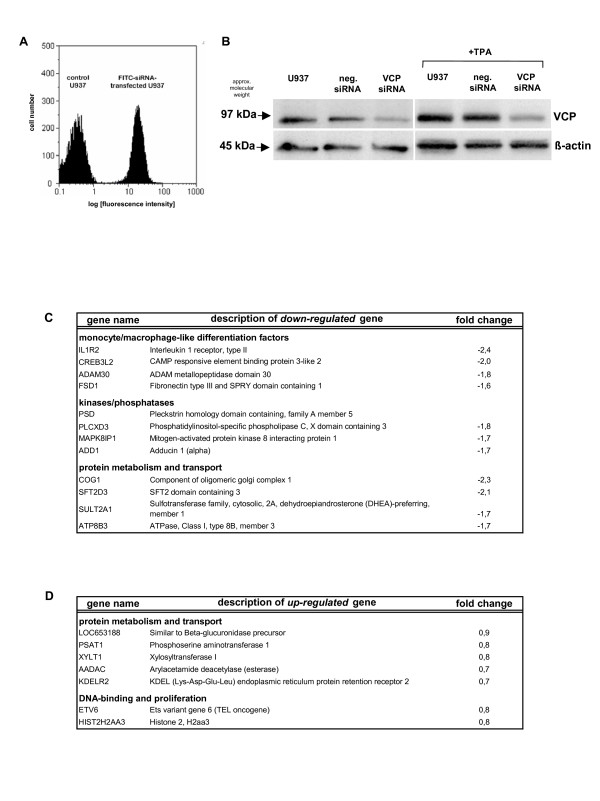
**siRNA transfection of U937 cells**. **A**. U937 cells (control U937) and cells transfected with a FITC-conjugated Negative Control siRNA AF488 (FITC-siRNA-transfected U937) were analyzed by FACS and revealed more than 95% transfection efficiency by this method. **B**. Western blot analysis of U937 control cells (U937), U937 cells transfected with a negative control siRNA (neg. siRNA) and U937 cells transfected with a VCP/p97 siRNA (VCP siRNA) were analyzed for VCP/p97 protein expression 72 h after the transfection and in the presence of 10 nM TPA for 24 h (+TPA), respectively. **C, D**. cDNA microarray analysis of VCP/p97 siRNA-transfected U937 cells during TPA-induced differentiation. U937 cells transfected with either negative control siRNA or VCP/p97 siRNA in the presence or absence of 10 nM TPA for 24 h, respectively, were subjected to cDNA microarray analysis provided by the Stanford Functional Genomics Facility. U937 control transfectants were compared to VCP/p97 transfectants and simultaneously TPA-treated control-transfectants were compared to the appropriate TPA-treated VCP/p97 siRNA transfectants to exclude the genes affected by the transfection itself. Thereafter, the regulated genes of untreated and TPA-treated populations were compared in order to evaluate those transcripts affected by VCP/p97 during the differentiation process. Potential off-target effects have been addressed by separate analysis of the two distinct VCP/p97 siRNAs in U937 and TPA-treated U937 cells, respectively. A representative panel of resulting down-modulated (C) and up-regulated (D) VCP/p97-affected genes during differentiation are demonstrated.

**Figure 7:**
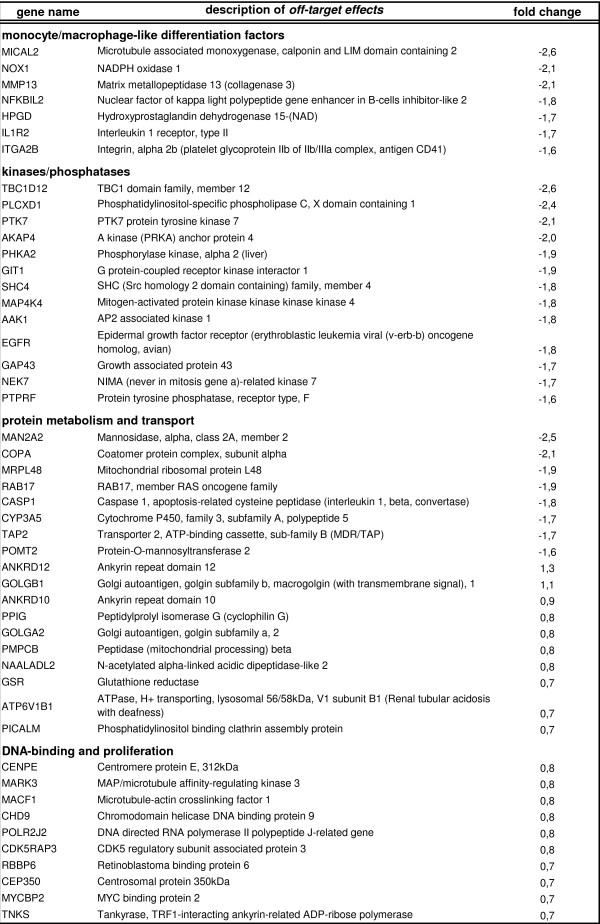
**Description of off-target effects during RNAi of VCP/p97**. Off-target effects reflect VCP/p97-siRNA-independent modulations of distinct genes. Only genes that were affected by both chosen VCP/p97-siRNAs were considered as down- or up-regulated.

A possible functional role of VCP/p97 in differentiating U937 cells was addressed by a siRNA approach. The transfection method was tested with a FITC-conjugated control siRNA which revealed a transfection efficiency in U937 cells of >95% as evaluated by FACS analysis (Fig. 6A). The successful down-modulation of VCP/p97 protein in VCP-siRNA-transfected cells was demonstrated in appropriate Western blots for both, U937 control and TPA-differentiating cells (Fig. 6B). Functional changes in VCP-siRNA-transfected cells following phorbol ester-induced monocytic differentiation have been examind by DNA microarray analysis (Fig. 6C, D). Thus, differentiation-associated genes, which are elevated during myelomonocytic maturation, including the cytokine receptor for interleukin-1, were expressed at significantly reduced levels in TPA-treated VCP/p97-siRNA-transfected U937 cells. According to the cell attachment as a consequence of differentiation along the monocytic/macrophage-like pathway, genes associated with the formation of the cytoskeleton (e.g. adducin1) and certain extracellular matrix related genes such as ADAM metalloproteinase 13 and the extracellular glycoprotein fibronectin showed a decreased expression due to VCP/p97 RNAi (Fig. 6C). Moreover, previous work has demonstrated that TPA-mediated signals in U937 cells are relayed via activation of protein kinase C and downstream kinases to activate appropriate transcription factors for the induction of differentiation-associated genes [8]. In this context, pleckstrin homology domain containing proteins, a MAP kinase interacting protein and phospholipase C were significantly down-modulated in TPA-treated VCP-siRNA-transfectants (Fig. 6C). With respect to genes involved in protein metabolism and particularly in transport functions, sulfotransferase 2A, an ATPase (ATP8B3), a vesicle transport protein (SFT2D3) and a Golgi-associated gene (COG1) were reduced expressed (Fig. 6C). In contrast, the KDEL receptor was up-regulated, which is responsible for the retention of newly synthesized proteins during the quality control in the ER [32] (Fig 6D). Moreover, metabolic compounds involved in post-translational modifications in the lysosome and microsome such as β-glucoronidase, arylacetamide deacetylase, phosphoserine aminotransferase and xylosysltransferase were increased expressed (Fig. 6D). TPA-induced differentiation is also associated with growth arrest and down-modulation of cell cycle-regulatory factors [9,10]. In contrast, TPA treatment of VCP-siRNA-transfected U937 cells revealed an increased expression of the transcriptional regulator ETV6 and certain histones. (Fig. 6D). However, siRNA-targeting in cells also involves off-target effects, which reflect VCP/p97-siRNA-independent modulations and some of these genes are listed in Figure 7.

In summary, the siRNA-targeted down-modulation of VCP/p97 in differentiation-induced U937 cells revealed a reduction of differentiation-associated genes and elevated levels of DNA-binding and proliferation-related factors.

## Discussion

Although the AAA ATPase VCP/p97 is involved in a variety of pathophysiological processes, providing a potential therapeutic target, the knowledge about its signaling cascades remain scarce. Previous studies suggested an unaltered expression of VCP/p97 throughout the cell cycle [14,17]. The results of the present study are in agreement with these findings. Moreover, an enhanced VCP/p97 phosphorylation of the yeast homolog Cdc48 was observed during spindle disassembly [15]. Cdc48 mutations revealed the requirement of this ATPase for the nuclear division process [19]. However, the role of VCP/p97-associated phosphorylation patterns for regulatory processes within the cell cycle remains unknown.

Cell cycle arrest in G_0_/G_1 _by TPA induction was associated with a pronounced VCP/p97 accumulation both, in the membranes and in the nucleus, which might be related to enhanced protein turnover according to the functional restructure of U937 cells during the concomitant differentiation program. Supportive findings from recent work indicated an involvement of VCP/p97 in transcriptional regulation [13] as well as in proteasomal degradation during differentiation. This effect was accompanied by a membrane-association of VCP/p97 required for retrotranslocation of misfolded and unwanted proteins to the cytosol with subsequent degradation by the ubiquitin-proteasome system [33]. Although previous work has suggested that elevated VCP/p97 expression is associated with increased metastatic potential and tumor recurrence in a variety of cancers, including hepatocellular, colorectal and osteosarcomal carcinoma [27-30], the present findings demonstrate an up-regulation of VCP/p97 during differentiation and growth arrest. This differentiation process is accompanied by the development of secretory active cells requiring significant endoplasmic reticulum-associated protein degradation [34,35] and thus VCP/p97 [36-38]. Previous data revealed that a down-modulation of VCP/p97 results in a significant accumulation of ubiquitinated and thus degradation-designated proteins, which eventually promotes apoptosis [39]. This is substantiated by microarray studies in HeLa cells transfected with VCP-siRNA, demonstrating a significant up-regulation of transcripts encoding for proteins associated with apoptosis, ER stress and oxidative stress, respectively [40]. Together, the findings may be limited to possible off-target effects due to sequence-independent cellular response to the siRNAs used.

The functions of VCP/p97 may contribute to the retrodifferentiation process with certain levels of nuclear-associated VCP/p97 before retrodifferentiation. Indeed, other work has demonstrated a simultaneous PARP-1-regulated activation of the nuclear proteasome with maximal proteolytic activity at the time point of retrodifferentiation [41,42]. Whereby inhibition of either PARP-1 or the proteasome blocks the retrodifferentiation program [42], these effects substantiate the importance of an intact proteasomal regulation by factors such as VCP/p97 to restore the undifferentiated U937 phenotype.

The requirement of VCP/p97 within the nucleus may also involve other regulatory functions for the retrodifferentiation program, which concludes the transition of a growth-arrested differentiated population to autonomously proliferating undifferentiated tumor cells. Such nuclear function was demonstrated for the interaction of VCP/p97 with DNA unwinding factor (DUF) in nuclei of Xenopus egg extracts and suggested a role in DNA replication [43]. As a contribution to a tumor development, VCP/p97 has been demonstrated to directly bind to BRCA1 as one of the key regulatory genes in breast cancer pathogenesis [13]. Since BRCA1 is involved in DNA damage repair, the interaction with the ATP transporter VCP/p97 altered the transcription-associated DNA repair [13]. Together, these regulatory processes suggest a tightly controlled subcellular distribution of VCP/p97 according to the appropriate cellular requirements. However, the signals for VCP/p97 to be directed to distinct subcellular compartments remain unclear. In this context, it is interesting to note that tyrosine phosphorylated VCP/p97 was exclusively associated with the cytosol and the membrane fraction, indicating a possible role for compartmentalization. This is supported by previous findings which suggested a possible role for subcellular localization of VCP/p97 following tyrosine phosphorylation [15,22]. Finally, these effects are accompanied by the involvement of VCP/p97 in the TPA-mediated monocytic transition, affecting the expression of differentiation-associated and cell cycle-regulatory genes. Moreover, genes involved in the ER-associated metabolic pathway were expressed at a significantly reduced level, whereas expression of genes implicated in post-translational modifications in the lysosomes and microsomes was considerably increased, indicating a support and substitution of the protein biosynthesis and quality control in the ER. This is further substantiated by the enhanced expression of the KDEL ER protein retention receptor [32].

## Conclusion

Taken together, the present findings suggest that VCP/p97 expression is not altered during proliferation and cell cycle progression. However, significantly increased protein and RNA levels appeared during G_0_/G_1 _cell cycle arrest and an associated differentiation process. A certain redistribution of VCP/p97 was observed in the membrane and the nuclear fractions, carrying a tyrosine phosphorylated form predominantly in the cytosolic and the membranous compartments. The altered expression levels of VCP/p97 during the TPA-induced differentiation of U937 cells as well as the changes of certain differentiation-associated transcripts as a consequence of VCP/p97 RNAi suggested that this AAA ATPase accompanies the differentiation and retrodifferentiation process.

## Methods

### Cell culture

Human U937 myeloid leukemia cells were grown in RPMI 1640 medium containing 10% fetal calf serum (Biochrom, Hannover, Germany) supplemented with 100 units/ml penicillin, 100 μg/ml streptomycin and 2 mM L-glutamine (PAA Laboratories GmbH, Cölbe, Germany). The cells were treated with 10 nM TPA (Sigma, St. Louis, MO, USA) for up to 72 h. The long term culture of adherent TPA-differentiated U937 cells was performed in the absence of TPA by changing the culture medium every 3 days until the cells entered the retrodifferentiation program.

### Centrifugal elutriation

The centrifugal elutriation was performed using the Beckmann J6-MC with the JE-5.0 rotor and the appropriate 5 ml-standard elutriation chamber (Beckman Coulter, Krefeld, Germany). Approximately 10^9 ^U937 cells were harvested, resuspended in PBS and applied to the standard chamber (1600 rpm at 24°C) using a digital flow controller (Cole-Palmer Instruments Inc., Chicago, IL, USA). Subsequent fractions of 100 ml aliquots of the elutriated samples were collected upon progressive increase of the pump speed. Elutriated cell fractions were examined for cell cycle distribution.

### Cell cycle distribution by FACS analysis

About 2 × 10^6 ^cells of the elutriated fractions were analyzed for distinct cell cycle phases as described previously [11]. Briefly, the cells were harvested and fixed in 70% (v/v) ice-cold ethanol and kept at 4°C for 24 h. Thereafter, the fixed cells were stained with CyStain DNA 2 step kit (Partec GmbH, Münster, Germany) and filtered through a 50 μm filter. These samples were then analyzed in a Galaxy flow cytometer (Dako, Hamburg, Germany) using FloMax analysis software (Partec) and the MultiCycle cell cycle software (Phoenix Flow Systems Inc., San Diego, CA, USA).

### RNA isolation

About 5 × 10^6 ^cells were lysed in 2 ml TRIZOL Reagent (Invitrogen, Karlsruhe, Germany) and incubated for 5 min at room temperature. After addition of 400 μl chloroform, the samples were mixed vigorously and then centrifuged for 15 min/12000 rpm at 4°C. An aliquot of 350 μl of the aqueous upper phase was incubated for 5 min at 25°C and treated according to the RNeasy Mini Kit protocol (Qiagen, Hilden, Germany). To determine the RNA concentration and integrity, the isolated RNA was analyzed in the Agilent 2100 Bioanalyzer (Agilent Technologies, Böblingen, Germany) using the RNA 6000 Nano LabChip Kit (Agilent Technologies) according to the manufacturer's protocol.

### RT-PCR

Reverse transcription was performed according to the RevertAid First Stand cDNA Synthesis protocol with Random Hexamer Primer (Fermentas GmbH, St. Leon Rot, Germany) using 1000 ng of DNAse-treated RNA. An aliquot of about 100 ng cDNA was amplified by quantitative RT-PCR in the LightCycler 2.0 System using the Light Cycler Software 3.5 (Roche Applied Science, Mannheim, Germany) according to the LightCycler FastStart DNA Master^PLUS ^Hybridisation Probes protocol (Roche). The TATA-binding protein, TBP, was used as a reference. For amplification, one cycle of 95°C for 15 min and then 40 cycles of 94°C for 15 s, 55°C for 25 s and 72°C for 25 s were performed with VCP forward primer 5'-TTCCTGAAGTTTGGCATGACAC and reverse primer 5'-GCGGGCCTTGTCAAAGAT as well as TBP forward 5'-TTCGGAGAGTTCTGGGATTGTA and reverse primer 5'-TGGACTGTTCTTCACTCTTGGC, respectively (TIB MOLBIOL, Berlin, Germany). The following hybridisation probes were used: VCP 5'-LC-Red640-CACCATGTGGTTTGGGGAGTCTGAG – PH and 5'-CTCCATCAAGGGTCCTGAGCTGC – FL and for TBP 5'-LC-Red640-TGAGGATAAGAGAGCCACGAACCACG – PH and 5'-CCAAGCCGGTTTGCTGCGGTAATC – FL. The RT-PCR quantification was performed using the RelQuant software (Roche).

### Immunocytochemistry

For immunofluorescence, the differentiation/retrodifferentiation process was performed on cover slips. The differentiated cells became adherent to the glass surface whereas U937 and retrodifferentiated cells were sedimented on poly-L-lysine coated slides. The cells were fixed with 4% (v/v) paraformaldehyde for 5 min at room temperature. After neutralisation with 1 × TBS and permeabilization with 0.1% (v/v) Triton X-100 in PBS, the slides were incubated with an anti-VCP antibody (Santa Cruz Biotechnology, Santa Cruz, CA, USA) in 3% BSA/PBS for 1 h at 37°C. After washing with PBS, the slides were incubated for 1 h at 37°C with a FITC-conjugated secondary antibody (Dianova, Hamburg, Germany) diluted 1:1000 in 3% BSA/PBS. Following an additional washing step, further incubation was performed with the DNA-intercalating dye Hoechst33258 (Invitrogen) for detection of the nuclei. After two final washing steps with PBS, the slides were mounted using ProLong Antifade (Invitrogen).

Additionally, the specificity of the VCP antibody was tested in a neutralization reaction. Therefore, the VCP antibody was preabsorbed with the appropriate specific VCP peptide (Santa Cruz Biotechnology) according to the manufacturer's protocol. The prepared slides were incubated as described above for 1 h at 37°C using this antibody-peptide-mixture instead of the primary antibody.

Epifluorescence microscopy was performed with a Zeiss Axiovert 200 M microscope using the Zeiss Axiovision software (Carl Zeiss, Göttingen, Germany).

### Separation of proteins from subcellular compartments

Separation of the different cellular compartment proteins was performed using the Qproteome cell compartment kit (Qiagen, Hilden, Germany). About 1 × 10^7 ^cells were harvested and lyzed according to the Qiagen protocol. All buffers were supplemented with protease inhibitors (according to the Qiagen protocol) and 1 mM NaVO_3 _as phosphatase inhibitor. The obtained proteins of the subcellular compartments were concentrated and desalted by acetone precipitation. Thereafter, the pellets were resupended in the appropriate buffer depending on further analysis.

### Immunoprecipitation

The immunoprecipitation was performed by the μMACS Protein G-MicroBeads method (protocol according to the manufacturer's recommendation; Miltenyi Biotech, Bergisch Gladbach, Germany). Briefly, the acetone-precipitated proteins were resuspended in 1 ml lysis buffer pH 7.5 containing 20 mM Tris, 150 mM NaCl, 1 mM EDTA, 1 mM EGTA, 2,5 mM NaPP_i_, 1 mM β-glycerolphosphate, 1 mM NaVO_3_, 1 μg/ml leupeptin, 1% (v/v) Triton-X-100, 0.5% (v/v) Nonidet-NP40 and 1 mM PMSF. For preabsorption of non-specific binding proteins, 50 μl μMACS Protein G-MicroBeads (Miltenyi Biotech) were added to the protein homogenate of the appropriate subcellular compartment and incubated for 30 min at 4°C. Thereafter, the suspension was applied onto the prepared μ-Column (Miltenyi Biotech) within the magnetic field of the μMACS Separator (Miltenyi Biotech). The flowthrough was collected in a new reaction tube and reused for subsequent antibody-incubation. For the immunoprecipitation, 1 μg of anti-phosphotyrosine antibody (clone 4G10, Upstate Biotechnology, Lake Placid, NY, USA) or 1 μg of anti-VCP antibody (Novus Biologicals, Littleton, CO, USA), respectively, and 50 μl μMACS Protein G-MicroBeads were added to the protein homogenates. After incubation for 30 min at 4°C, the suspension was applied onto new μ-Columns and several washing steps with lysis buffer were performed according to the manufacturer's protocol (Miltenyi). Finally, a low salt buffer, containing 20 mM Tris-HCl pH 7.5, was added and elution was performed using an adequate buffer, e.g. 50 μl 1× SDS gel loading buffer (95°C) for subsequent SDS-PAGE.

### Two-dimensional (2D) polyacrylamide gel electrophoresis (SDS-PAGE)

The proteins of the different subcellular compartments were separated by isoelectric focusing (IEF) followed by SDS-PAGE in the second dimension, respectively. Thus, the proteins were incubated in reswelling buffer (8 M urea, 1% CHAPS (v/v), 0.5% pharmalytes 3–10 (v/v), 0,002% bromphenol blue (w/v), 0,4% DTT (w/v); according to the Amersham protocol) on an 18 cm IPG-Immobiline Dry Strip (pH 3–10; NL) (Amersham Biosciences GmbH, Freiburg, Germany) and separated for 18 h at 150 V in the first dimension using the IPGphor isoelectric focusing system (Amersham). Thereafter, the IPG strips were incubated in two subsequent equilibration buffers for 15 min, respectively (according to the Amersham protocol), and polymerised on a 10% SDS-PAGE separation gel using 0.5% (w/v) low melting point agarose. Electrophoresis was standardized using appropriate molecular-weight markers (Amersham).

### Immunoblot analysis

SDS-PAGE was performed in a 10% and 7.5% separation gel, respectively, and was standardized using prestained low-molecular-weight markers (Bio-Rad, Munich, Germany). Following protein transfer to nitrocellulose membrane filters (Amersham Bioscience GmbH, Freiburg, Germany), the blots were incubated with a monoclonal antibody against VCP/p97 (Progen Biotechnik GmbH, Heidelberg, Germany) and a monoclonal anti-phosphotyrosine antibody (clone 4G10, Upstate Biotechnology, Lake Placid, USA). Appropriate antibodies against selected subcellular compartment standard proteins such as anti-GAPDH (Santa Cruz Biotechnology, Santa Cruz, CA, USA) for the cytosol, anti-clathrin (Progen) for the membrane fraction and anti-nucleoporin62 (BD Pharmingen, San Diego, CA, USA) for the nuclear compartment were used to ensure an appropriate subcellular separation. Following incubation with a peroxidase-conjugated secondary antibody (Amersham), the blots were developed using an electrochemiluminescence (ECL) detection kit (Perkin Elmer, Boston, MA, USA).

### siRNA-transfection

Transfection of the cells was performed according to the manufaturer's protocol (amaxa GmbH, Cologne, Germany). Briefly, 1 × 10^6 ^U937 cells were harvested and resuspended in 100 μl Nucleofector Solution V (amaxa). The cell suspension was mixed with 1 μg siRNA, placed in a sterile electroporation cuvette and subjected to program T20 using the Nucleofector II (amaxa). For transfection, the following siRNAs were used: GW VAL siRNA Hs_VCP_6 and GW VAL siRNA Hs_VCP_7 to target VCP/p97, AllStars Negative Control siRNA as a negative control, and Negative Control siRNA AF488 as a FITC-conjugated negative control (all from Qiagen, Hilden, Germany), respectively. After transfection, the cells were immediately transferred into pre-warmed culture medium, supplemented with 10% FCS. To evaluate the transfection efficiency, U937 controls and cells transfected with Negative Control siRNA AF488 were harvested after 5 h, washed twice with PBS and subjected to the Galaxy flow cytometer (Dako) using FloMax analysis software (Partec). For VCP/p97 Western blot analysis, the transfected cells were harvested 72 h post transfection and treatment with 10 nM TPA was performed for 24 h.

### cDNA microarray analysis

For cDNA microarray experiments U937 cells were transfected either with negative control siRNA, the two VCP/p97 siRNAs together or each VCP/p97 siRNA separately, respectively. The differentiation process of these four populations was initiated 48 h after transfection by treating the cells with 10 nM TPA for additional 24 h. Control cultures were maintained in medium for 72 h after transfection. Following RNA extraction, 1 μg of total RNA was linearly amplified using the MessageAmp aRNA Kit (Ambion, Huntington, UK). Quantification and validation for integrity was performed on an Agilent 2100 Bioanalyzer using the RNA 6000 Nano LabChip Kit according to the manufacturer's protocol (Agilent Technologies, Böblingen, Germany). Three μg of amplified RNA from the different VCP/p97 siRNA transfected cells of both, the untreated and TPA-treated population, was labelled with Cy5-dUTP, whereas the amplified RNA from negative control siRNA transfected cells was similarly labelled with Cy3-dUTP according to the provided protocol (Amersham Biosciences), respectively. To evaluate off-target effects, we performed microarray analysis of pooled triplicates of transfected cells with each of the VCP/p97 siRNAs separately, and then compared these results to the microarray data of both VCP/p97 siRNAs together. The fluorescence-labelled RNA probes were comparatively hybridised on a spotted cDNA microarray (Stanford Functional Genomics Facility, Stanford, CA, USA) following the posted protocol [44]. The fluorescence intensities of Cy5 and Cy3 were measured on a GenePix 4000 scanner (Axon Instruments, Foster City, CA, USA) and analyzed using GenePix Pro 4.1 software (Axon Instruments). Areas of the microarray or spots exhibiting obvious damages due to technical failures were excluded from subsequent analysis. Single spots were only considered as well-measured and included in the further investigation, when the mean fluorescent intensity of Cy5 and Cy3 was ≥ 3-fold above the local background and the regression correlation was ≥ 0.8. Genes were classified as upregulated, when the corresponding spot met these quality criteria and a log (base 2) ratio of Cy5 relative to Cy3 revealed a value of ≥ 0.7. Thus, U937 control transfectants were compared to VCP/p97 transfectants and simultaneously TPA-treated control-transfectants were compared to the appropriate TPA-treated VCP/p97 transfectants to exclude those genes affected by the transfection itself. Thereafter, the regulated genes of untreated and TPA-treated populations were compared in order to exclusively evaluate transcripts affected by VCP/p97 during the differentiation process. Finally, these gene expressions were cleared by the off-target effects. All cDNA microarray expression data, including the off-target effects, are stored online with the accession number GSE9821 at the GEO website [45].

## List of abbreviations

valosin-containing protein (VCP/p97)

12-O-tetradecanoyl-phorbol-13-acetate (TPA)

## Authors' contributions

CB carried out the cell biological studies. BSk and DS contributed the DNA microarray data. NvN and RH participated in the design and coordination of the study. BS contributed in critical reviewing and revising of the manuscript. CB and RH drafted the manuscript.

All authors have read and approved the final version of this manuscript.
